# Exploring the contextual assumptions, interventions and outcomes of digital advance care planning systems: A theory of change approach to understand implementation and evaluation

**DOI:** 10.1177/02692163241280134

**Published:** 2024-09-21

**Authors:** Andy Bradshaw, Matthew J Allsop, Jacqueline Birtwistle, Catherine J Evans, Samuel D Relton, Suzanne H Richards, Maureen Twiddy, Robbie Foy, Pablo Millares Martin, Sarah Yardley, Katherine E Sleeman

**Affiliations:** 1 Cicely Saunders Institute of Palliative Care, Policy & Rehabilitation, King’s College London, London, UK; 2 Leeds Institute of Health Sciences, University of Leeds, Leeds, UK; 3 Hull York Medical School, University of Hull, Hull, UK; 4 Whitehall Surgery, Leeds, UK; 5 Marie Curie Palliative Care Research Department, University College London, London, UK

**Keywords:** Palliative care, electronic health record systems, advance care planning, end of life care, technology, mid-range programme theory

## Abstract

**Background:**

Digital advance care planning systems are used internationally to document and share patients’ wishes and preferences to inform care delivery. However, their use is impeded by a limited understanding of factors influencing implementation and evaluation.

**Aim:**

To develop mid-range programme theory to account for technological, infrastructure and human factor influences on digital advance care planning systems.

**Design:**

Exploratory qualitative research design incorporating Theory of Change workshops that explored contextual assumptions affecting digital advance care planning in practice. A mid-range programme theory was developed through thematic framework analysis using the Non-adoption, Abandonment, Scale-up, Spread and Sustainability (NASSS) framework, generating a conceptual model depicting contextual assumptions, interventions and outcomes influencing implementation.

**Participants:**

A total of 38 participants (16 from London, 14 from West Yorkshire and 8 online) including patients, carers and health and care professionals (including those with commissioning responsibilities).

**Results:**

A conceptual model was generated depicting five distinct components relating to digital advance care planning system use: (sociocultural, technical and structural prerequisites; recognition of the clinical need for conversation; having conversations and documenting decisions; accessing, actioning and amending; and using data to support evaluation, use and implementation). There were differences and uncertainty relating to what digital advance care planning systems are, who they are for and how they should be evaluated.

**Conclusions:**

Digital advance care planning lacks shared beliefs and practices, despite these being essential for complex technology implementation. Our mid-range programme theory can guide their further development and application by considering technological, infrastructure and human factor influences to optimise their implementation.

**What is already known about the topic?**

- Internationally, digital approaches for documenting and sharing advance care planning information are increasingly used in palliative and end-of-life care delivery.
- Digital advance care planning systems are becoming an integral component of palliative and end-of-life care policy, but there have been challenges with their uptake and use in routine practice.
- There is a need for robust and theoretically informed evidence to understand how digital advance care planning systems are used in practice, their intended impacts and how best to optimise and evaluate their use.

**What this paper adds?**

- Our mid-range programme theory defines factors that influence digital advance care planning system implementation across five components (sociocultural, technical and structural prerequisites; recognition of the clinical need for conversation and digital advance care planning; having conversations and documenting decisions; accessing, actioning and amending; and using data to support evaluation, use and implementation) of their use in routine care.
- Across patients, carers and health and care professionals (including commissioners) there is a lack of clarity, and divergent views, on the purpose and intended impact of digital advance care planning systems, who they are for and professionals’ responsibilities relating to their use.
- Digital advance care planning systems lack shared practices essential for effectively implementing complex technological interventions and maximising their potential impact.

**Implications for practice, theory or policy**

- The five components of digital advance care planning system implementation presented in the conceptual model can support the development of defined outcomes and indicators of success important to system implementation.
- There is a need to consider how digital advance care planning systems interact with wider technological, infrastructure and human factors influences to guide their further design and implementation.

## Background

High-quality palliative care includes providing patients with the opportunity to discuss their wishes and preferences and coordinating services to deliver concordant care.^1–3^ Digital approaches are being developed internationally to support this process through documentation and sharing of patient information and preferences.^4–8^ Typically, digital advance care planning approaches involve creating a digital record of a person’s wishes and preferences that can be shared across settings involved in their care delivery.^9^ Digital advance care planning is often intended for people receiving palliative care, to support multidisciplinary working and care coordination.^9^ Digital advance care plans may include demographic information, diagnosis, medication and advance care planning information including resuscitation decisions and preferred places of care and death.^10^ Preliminary evidence has demonstrated the potential of such approaches to improve care quality by increasing the likelihood of patients achieving their preferred place of death and avoiding unplanned hospital admissions.^11–14^

While there is a growing international focus on digital advance care planning approaches in health and care policies, there has been variation in their functionality and implementation and a lack of clarity on how they might achieve impact.^3,4,15^ Multiple challenges, including poor interoperability across settings, exist with their use in practice.^16^ Furthermore, fewer than 1 in 10 people have a digital advance care planning record created before they die,^11,17–19^ often created close to the end of life.^11^ The development, implementation and evaluation of digital approaches to advance care planning have been hampered by a lack of a robust theoretical underpinning, concerns surrounding data privacy and inadequate consideration of the multiple stakeholders involved in their use.^16,20,21^ This study aimed to develop mid-range programme theory to account for technological, infrastructure and human factor influences on digital advance care planning systems.

## Methods

### Study design

An exploratory qualitative research design using theory of change workshops^22^ was used to explore technological, infrastructure and human factor influences on digital advance care planning. Theory of change approaches are one way of developing mid-range programme theory (i.e. a theory of how and why a programme or intervention works; mid-range indicates the theory has a level of abstraction from specific context while remaining close enough to empirical data to be used as an applied framework^23^), explicating the different ingredients (i.e. elements and contextual factors) required to improve the likelihood of achieving impact.^24^ The mid-range programme theory is developed iteratively with stakeholders and represented in a theory of change map (i.e. a graphical representation of what is required for its intended impact to be realised).^24^ We sought to develop a mid-range theory delimited to a specific application, to provide a framework for understanding and developing digital advance care planning.^25^ The study was developed with patient and public involvement representatives who contributed to the design and development of the study and advised on recruitment, participant information, consent materials and workshop design.

### Setting

Two regional workshops took place in England, in Greater London (workshop 1) and West Yorkshire (workshop 2), alongside an online event (workshop 3).

### Population

We sought to involve patients and informal carers with experience of palliative care, health and care professionals involved in palliative care delivery and clinical and non-clinical professionals with palliative and end-of-life care commissioning and service leadership responsibilities.

### Sampling

Patients and carer participants included those who accessed services for people with life-limiting or long-term conditions or non-governmental organisations that support people to make advance care plans. Health and care professionals were purposively sampled based on their professional role and included specialist palliative care (i.e. hospices and hospital teams) and community nursing, general practitioners, paramedics and care homes. We also sought the involvement of clinical and non-clinical staff with palliative and end-of-life care commissioning and leadership responsibilities.

### Recruitment

This was the final study in a five-phase project exploring the implementation of digital advance care planning systems.^16^ For workshops 1 and 2, we targeted participants who had taken part in earlier phases of the project, supplemented with recruitment of new participants from underrepresented groups. Recruitment to workshop 3 took place using commissioner networks across Yorkshire.^16^ Between January and May 2023, we emailed invitations to potential participants. All workshop attendees provided consent either written or via a secure online form.

### Data collection

#### Workshops 1 and 2

These in-person, 4-h workshops used the theory of change approach to explore how interactions between individuals, organisations and system-level contextual assumptions affect how and why digital advance care planning systems work in practice.^24,26^ In England, digital advance care planning systems are commonly called Electronic Palliative Care Coordination Systems (abbreviated to EPaCCS).^8^ Consistent with a collaborative approach, participant groups (patients, informal carers, health and care professionals) were mixed across three tables, with 4 – 6 participants (ensuring representation of each participant group) at each table accompanied by two experienced research team members (a facilitator and a scribe). Four activities were completed: (1) discussion of intended impacts and outcomes of EPaCCS, (2) prioritisation of impacts and outcomes, (3) development of a theory of change map, sequentially working backwards from identified outcomes to consider the preconditions required to achieve them and (4) circulation between tables to discuss the emergent theory of change maps. The structure and overall facilitation of workshops 1 and 2 were led by a research team member, acting as a theory of change champion (SY). Event artefacts (see Appendix A) generated across activities (e.g. notes taken by scribes, sticky notes, flipcharts, cards, photographs and researcher field notes) were treated as data for analysis.

#### Workshop 3

Preliminary findings from earlier workshops informed the four areas pursued during this online event (via Microsoft Teams): (1) the purpose and intended outcomes of EPaCCS (e.g. with whom EPaCCS should be used); (2) how EPaCCS differ from other plans that may have relevance in end-of-life situations (e.g. Recommended Summary Plan for Emergency Care and Treatment [ReSPECT]^27^) and EPaCCS relevance to end-of-life policy and commissioning priorities; (3) challenges experienced when implementing EPaCCS; and (4) future research priorities. Whole group discussions took place, followed by two smaller breakout rooms which were facilitated by members of the research team (KS and MA). The meeting lasted 90 min with discussions auto-transcribed by Microsoft Teams, then checked and anonymised by the research team.

Across all workshops, at least seven research team members remained constant, facilitating and participating in discussions to support continuity. In all workshops, disagreement among participants was explored through discussion.

### Data analysis

**Figure 1.**
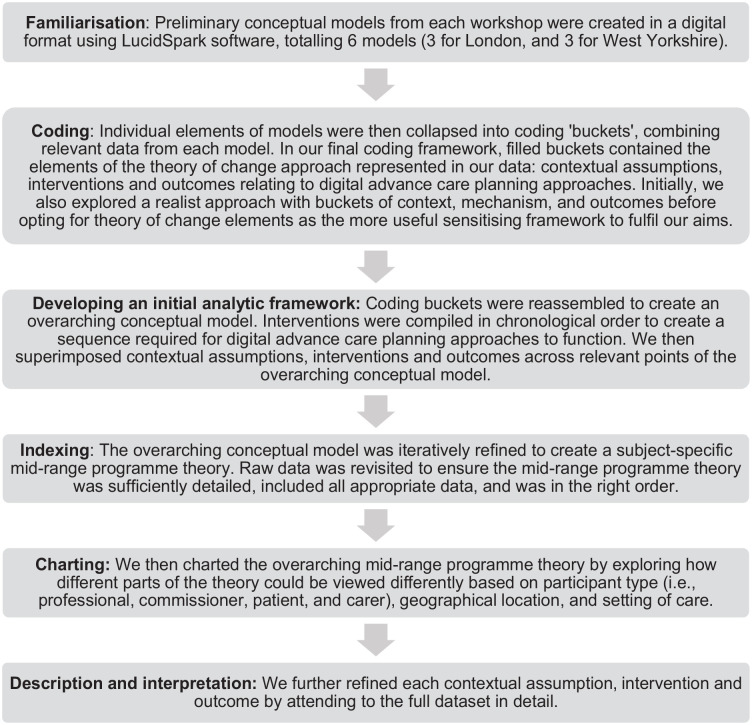
Steps followed during thematic framework analysis.

We adopted a pluralistic approach to data analysis.^28,29^ Using different, but mutually enriching, analytic techniques enabled us to explore the complexity and nuance across the datasets.^30^ Initially, we used thematic framework analysis (Figure 1) to develop an overarching conceptual model based on conceptual models generated during workshops and refined using workshop discussion notes.^31^ The preliminary conceptual model was a diagrammatic representation of the mid-range programme theory, describing the contextual assumptions, interventions and outcomes influencing EPaCCS implementation, alongside areas of uncertainties surrounding these.^32^ Thematic framework analysis allowed an overarching synthesis of all data, but could not capture the granularity, nuance (‘real life’ messiness) and divergence within the data. This was evident when we started to chart, describe and interpret data (steps 5 and 6, Figure 1). To embrace these complexities, we engaged in ‘thinking with theory’, drawing on multiple theoretical lenses to produce different knowledge and ways of thinking.^33,34^ We identified the Non-adoption, Abandonment, Scale-up, Spread and Sustainability (NASSS) framework^35^ as offering explanatory power that supported the development of a rich and situated narrative, accommodating data from conceptual models, scribe discussion notes and researcher field notes. The NASSS framework consists of seven domains (health condition, the technology, the value proposition, the adopter system, the organisation(s), the wider system and changes over time). It was used deductively to categorise and explain the nuances and divergences in views regarding different elements of our conceptual model. Our conceptual model evolved iteratively through multiple research team discussions over two months, drawing on findings and experiences from earlier phases of the research project,^16,36,37^ and combining findings aligned to the NASSS framework. From this, we developed an initial mid-range programme theory. This sought to convey the contextual assumptions, interventions and outcomes of EPaCCS.^22,38^

We adopted a relativist approach to rigour.^39^ We used contemporaneous methodological discussions on rigour and lists of quality criteria and techniques.^40^ We made informed choices in selecting criteria and techniques that were most applicable to the context, aims and design of this study^39,41^ (Appendix B lists quality criteria and how they were met). A sub-sample of four patient and carer participants acted as ‘critical friends’ during analysis, discussing developing analyses, providing alternative interpretations of the data and ensuring the conceptual model (representing our mid-range programme theory) was an accurate reflection of participants’ accounts.

## Results

Across the three workshops, we recruited 38 participants (Table 1). In workshops 1 and 2, 53.3% of recruits had participated in the preceding study phases.

**Table 1.** Participant demographic information by workshop.

Sample characteristics	1: London *N* (%)	2: West Yorkshire *N* (%)	3: Online *N* (%)
No. of attendees	16	14	8
Professionals (by setting)			
Hospice	4 (25)	3 (21)	–
Nursing/residential care home	2 (12.5)	1 (7)	–
Hospital	2 (12.5)	1 (7)	1 (12.5)
Community	1 (6.3)	3 (21)	2 (25)
Primary care	1 (6.3)	–	1 (12.5)
Ambulance	1 (6.3)	1 (7)	–
Integrated care board (ICB)	–	2 (14)	4 (50)
Community + hospital	–	1 (7)	–
Patients (by diagnosis)			
Cancer	2 (12.5)	-	–
Multiple long-term conditions	–	2 (14)	–
Carers (by role)			
Bereaved carer	2 (12.5)	–	–
Charity organisations	1 (6.3)	–	–

### Mid-range programme theory

**Figure 2.**
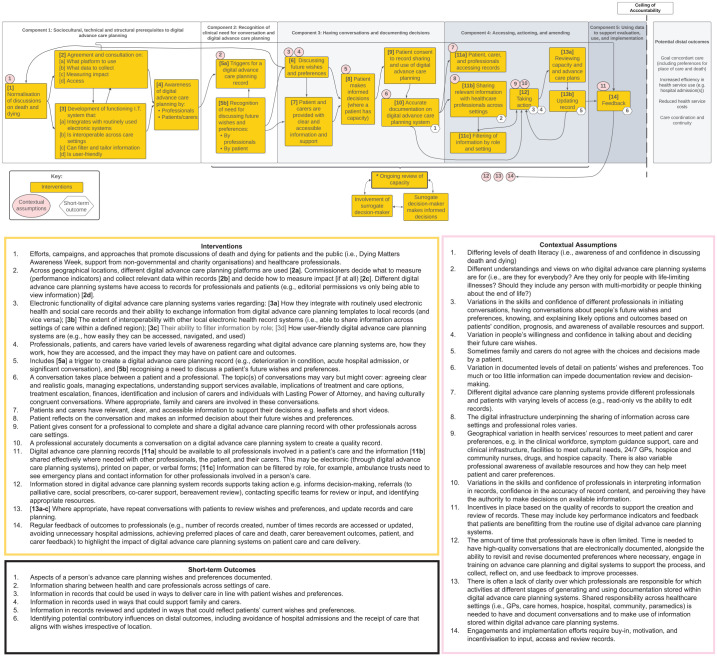
Mid-range programme theory depicting the contextual assumptions, interventions and outcomes influencing digital advance care planning implementation.

A mid-range programme theory is presented in Figure 2, providing a graphical representation of the different elements required for digital advance care planning systems to achieve their short-term impact. The mid-range programme theory comprises five components: sociocultural, technical and structural prerequisites; recognition of clinical need for conversation and digital advance care planning; having conversations and documenting decisions; accessing, actioning and amending; and using data to support evaluation, use and implementation.

### Alignment with the NASSS framework

We aligned our findings with the domains of the NASSS framework (Table 2).

**Table 2.** Workshop data aligned with NASSS framework domains to provide an overview of participant perspectives.

Relevant NASS framework components and sub-components	Definition	Key findings from the workshop data
1. Health condition		
(a) Nature of condition	Clinical aspects of patients’ condition(s), including its complexity (simple and predictable illness trajectory vs unpredictable and poorly understood) alongside who is deemed ‘suitable’ for the technology.	There were varied views regarding who participants thought EPaCCS were intended for. These ranged from people with palliative care needs to anybody who wanted to document preferences for care. Commissioners recognised that the use of EPaCCS for people with different disease trajectories and prognoses is likely to vary, but that this had seldom been considered in their design and implementation. Patients and carers considered that documenting future wishes and preferences can empower people to take control of their care.
(b) Socio-cultural factors and co-morbidities	The relevant sociocultural factors and comorbidities that affect care.	Normalising societal discussions of death and dying, ensuring EPaCCS templates were culturally congruent and addressing inequalities in access to palliative care were seen as important to consider when developing and implementing EPaCCS. Participants also highlighted the need to appreciate and address how EPaCCS intersects with broader inequalities in access to and engagement with palliative and end-of-life care.
2. The technology		
(a) Key features	The material and technical features of EPaCCS (e.g. size, sounds, aesthetics and user experience of interaction), and how these impact actual and perceived usability, appropriateness and dependability.	There were varied views across participants about the defining features of EPaCCS, how these were intended to function and who should access records (and to what extent).
(b) Types of data generated	The knowledge and data are generated/made visible by EPaCCS, and the extent to which those data are accepted, trusted and considered sufficient for decision-making.	A range of opinions were present across participants regarding the type, depth and detail of information that they thought should be documented within EPaCCS records. Detailing broader cultural, spiritual, personal and non-clinical preferences (e.g. dietary requirements) within records was thought to be helpful for professionals to understand the patient as a person, potentially in instances where they may not be able to communicate.
(c) Knowledge needed to use	Knowledge and support needed to use EPaCCS, whether this be from staff, patients and/or carers	Patients, carers and professionals needed to initially be aware of the existence of EPaCCS. Patients and carers suggested roles for non-governmental and charity organisations in raising awareness of EPaCCS and supporting advance care planning documentation.
3. The value proposition		
(a) Supply-side value (to developer) AND (b) Demand-side value (to patient and others)	Upstream value of EPaCCS for those who develop EPaCCS The desirability, efficacy, safety and cost-effectiveness of EPaCCS.	Across and within participant groups, there was diversity in opinions on what EPaCCS are, who they are for and their purpose, alongside a range of perspectives on how their impact could be measured.
4. The adopter system		
(a) Staff	What needs to be done in adopting and continuing to use EPaCCS? Or, alternatively, factors that lead towards non-adoption or abandonment. These may include whether the technology fits within their scope of practice or safety/welfare/benefits for patients.	EPaCCS requires professionals to recognise the need for and facilitate an advance care planning conversation. However, there was a lack of clarity over which professionals were supposed to engage with which aspects of EPaCCS and when.
(b) Patient and (c) carers	The work patients are expected to do in using EPaCCS What assumptions are there about the availability of carers and behaviours of carers?	Despite wanting to contribute to using, developing and designing EPaCCS, patients and carers felt opportunities to do this were limited.
5. The wider system		
(a) Economic and political context	The wider political and economic professional and socio-cultural context of EPaCCS	All participants groups agreed that it was important for the development and implementation of EPaCCS to be underpinned by proportionate funding, investment and long-term government commitment.
(b) Regulatory context	The wider regulatory, professional and socio-cultural context of EPaCCS	Data protection laws and safeguarding were seen as crucial to consider in the development and use of EPaCCS. However, participants were not always clear on the legal or regulatory implications of EPaCCS.
6. Embedding and adaptation over time		
(a) Scope for adaptation over time	The medium to long-term feasibility of continuing to adapt EPaCCS and its implementation within a service.	Sustaining the implementation of EPaCCS was viewed as an inherently ongoing and gradual process that required continual effort. This, alongside how to best involve patients, carers and professionals, needs to be considered when adapting EPaCCS to local contexts.

#### Health condition

##### Nature of condition

Participants had divergent views on who EPaCCS are intended for. Commissioners and professionals generally saw EPaCCS as intended for people with life-limiting illnesses and/or palliative care needs, regardless of prognosis. Patients and carers had varied and broader views on who EPaCCS are intended for and included people with severe mental health conditions and those with long-term physical conditions (e.g. people with sickle cell disease). Some patients, carers and professionals suggested that EPaCCS were relevant for anyone who wanted to document their preferences for care (regardless of any diagnosed illnesses) and supported an ‘opt-out’ approach in which everybody is given an EPaCCS unless they state an explicit preference not to have one.

##### Sociocultural factors

Participants broadly agreed that normalising discussions of death and dying (in society and across health and care) was important in supporting relevant discussions and EPaCCS implementation. Some patients and carers suggested that diverse community groups could play a role by providing information on EPaCCS at critical points in people’s lives (e.g. when creating advance funeral plans, writing a will and recording Lasting Powers of Attorney). Commissioners highlighted the importance of ensuring underserved groups can have their preferences discussed, documented and acted on. Patients and carers highlighted the need for EPaCCS templates to be culturally congruent by including information on cultural and religious elements of their care.

#### The technology

##### Key features

The meanings that participants attributed to EPaCCS and their intended purpose varied markedly. For some professionals, EPaCCS represented an electronic form that simply replaced paper-based records. For others, EPaCCS had the potential to transform how professionals communicate with and share information about patients. This lack of clarity contributed to difficulties in differentiating EPaCCS from other initiatives (e.g. ReSPECT^42^ documentation, and disease-specific care plans). Most participants agreed that EPaCCS should be accessible to all professionals involved in the care of patients (including general practitioners, community nurses and allied health professionals, social care staff, palliative care teams, hospital teams and paramedics). Patients and carers wanted access to their EPaCCS record. However, views varied on the level and type of information that should be accessible to carers and the extent to which records should be editable by patients.

##### Types of data generated

There was a range of opinions regarding what data should be documented within an EPaCCS record. Patients and carers were concerned that records could be overly focussed on physical (condition and symptoms), medical (e.g. medications, treatment plans and DNACPR decision) and clinical (e.g. contact information for professionals and services involved in care, preferred place(s) of care and death) aspects of care. They felt information within a record should be detailed and holistic, including broader cultural, spiritual, personal and non-clinical preferences (e.g. dietary requirements). Including caregiver information in records was deemed important but there were concerns regarding what and how much information about the caregiver could be included. In contrast, professionals (including paramedics) considered that records should be concise, enabling fast access to information in an emergency.

##### Knowledge needed to use

Health and care professionals, patients and carers must understand what EPaCCS are, how they work, how they can be accessed, how to use them (e.g. document, revise, update and share information) and potential impacts on patient care and outcomes. Patients and carers suggested roles for non-governmental and charity organisations in raising awareness of EPaCCS.

#### The value proposition

Demonstrating the benefits of EPaCCS on patient care was considered essential for health and care professionals to dedicate the necessary time and resources required to use them. However, there were mixed perspectives between and among patients, carers, professionals and commissioners on what the intended benefits of EPaCCS were, for whom and how their impact could be measured. Commissioners saw EPaCCS as a tool for ‘soothing the system’ and managing resources, including reducing emergency hospital admissions to relieve pressures on the acute system. Health and care professionals identified additional benefits, including prompting conversations on wishes and preferences, providing a template to guide advance care planning and informing decision-making in crises and emergencies. Patients and carers spoke of the value of EPaCCS in supporting personalised goal-centred care, as opposed to its impact on service delivery.

#### The adopter system

##### For staff

For professionals, ‘wrap-around’ work (i.e. activities related to, but not directly involving, EPaCCS) is needed for EPaCCS to impact on patient outcomes. This includes having conversations about preferences for care (as in Figure 1). Participants suggested that any professionals seeing patients for routine, urgent or inpatient care should be able to access and edit records. Ensuring access for all professionals was seen as a way of reducing duplication of effort and flexible use of systems (e.g. enabling emergency services access to view records and alert other health and care professionals to review a record).

##### For patients and carers

There were disconnects within the patient and carer participant group about what they were permitted to contribute within the current system. All patients wished for access to their own EPaCCS records. All believed that patients should have access to their own EPaCCS record. For some, this was limited to viewing their records, while others wanted to be able to edit their preferences. Many patients wanted to use EPaCCS to trigger conversations with professionals by requesting appointments to discuss, review and update their preferences. Patients and carers wanted to be involved in the future development of EPaCCS (for example, through patient and public involvement). This included being consulted on information contained within records and access.

#### The wider system

##### Economic and political context

All participant groups highlighted that long-term investment was needed to raise public and professional awareness of EPaCCS, further develop information technology systems so that they are functional (i.e. accessible, interoperable and easy to use), and train professionals in integration of EPaCCS within routine care. Some participants felt a national (rather than piecemeal) solution – that is, a universal EPaCCS platform that works across all care settings – was needed. Initiatives to increase professional engagement (particularly non-palliative care professionals) in advance care planning and use of EPaCCS were proposed.

##### Regulatory context

Participants recognised the importance of legal, ethical and regulatory frameworks governing data collection and protection, documentation, levels of access, data ownership and data security. However, participants across all groups were uncertain about the legal status of EPaCCS records (i.e. if the information within records was bound to any laws). Commissioners queried how EPaCCS related to and differed from other end-of-life plans (e.g. living will, ReSPECT planning documentation and Lasting Power of Attorney). Some professionals expressed concern about their lack of clarity regarding the legal and regulatory implications of EPaCCS.

#### Embedding and adaptation over time

##### Scope for adaptation over time

There was an appreciation, especially amongst commissioners, that promoting professional engagement with EPaCCS required considerable effort in return for slow shifts in clinical practice and culture. Geographical differences in types of EPaCCS led to suggestions for locally tailored implementation plans. These could adapt how EPaCCS are used when changes in organisations are made (e.g. during the introduction of new services), and being able to extract and use data from EPaCCS records to monitor and improve care. Integral to the adoption of EPaCCS over time was professional, patient and carer engagement to understand what works, what does not and what needs to change for EPaCCS to function better for each of these groups.

## Discussion

### Main findings

This study contributes a mid-range programme theory that outlines contextual assumptions, interventions and outcomes influencing the implementation of digital advance care planning systems. The theory draws upon the NASSS framework and captures granular and divergent views of patients, carers, health and care professionals and commissioners to enrich understanding of digital advance care planning system implementation, including the technological infrastructure and human factor influences. Participants from professional groups understood the value of digital advance care planning systems primarily in terms of service delivery and clinical decision-making, including achieving the preferred place of care and death, reflecting existing research and policy.^12^ Patients and carers considered additional, broader, aspects such as symptom management, not receiving unwanted medical interventions, family and carer support (including through bereavement), maintaining social ties and achieving a ‘good death’.

### What this study adds?

We identify five components of digital advance care planning that can support implementation and evaluation. The proximal, process-related outcomes may be simpler to quantify (e.g. whether a record is documented or updated, and how many times a record is accessed). However, we are reticent to hypothesise links between proximal process-related outcomes and more distal outcomes related to care quality (e.g. achieving a preferred place of care or death, delivering care in line with wishes and preferences, avoidance of hospital admissions). This aligns with earlier theory of change approaches exploring advance care planning in nursing homes, similarly narrowed to proximal outcomes.^43^

Digital advance care planning approaches are an emerging area of practice internationally.^8^ Further research that determines whether digital advance care planning systems are effective in achieving patient-centred outcomes is essential. The widespread variation and unstructured evolution of such systems^16^ may contribute to divergence in views on their purpose and intention. Changing work practices and evolving technology systems could create a context in which unintended consequences (e.g. care delivered that does not align with a person’s wishes due to inaccessible, outdated or inaccurate information) arise.^44^

### Strengths and weaknesses

This study is the first to integrate the perspectives of patients and carers, alongside healthcare professionals and commissioners, to understand the contextual assumptions, interventions and outcomes that influence the implementation of digital advance care planning approaches. Participants were recruited across two major regions of the UK, representing a diverse range of experiences as well as several different digital advance care planning systems. Our novel pluralistic approach adopts a theory of change approach, supplemented by the NASSS framework. Approaches combining a theory of change approach with complementary theoretical frameworks have been used previously to develop palliative care-based interventions.^45^ We chose a theory of change approach over alternatives such as realist evaluation, we found it was better suited for explicating implementation theory for the purpose of improvement and development of robust monitoring at a macro programme level.^46^ Our mid-range programme theory provides a novel understanding of digital advance care planning, alongside the complexities, nuances and conflicts regarding their implementation. The depth and detail of study findings provide a strong foundation for naturalistic generalisations to be made to other contexts in which digital advance care planning approaches are being used (e.g. MyHealthRecord in Australia and online patient portals in the USA^47^). However, we recognise that programme theory is developed iteratively, and the existing mid-range programme theory may be further refined in future work to incorporate additional elements, including indicators and rationale, and greater linkage of intervention activities and outcomes.

Study findings were reviewed and discussed with a sub-sample of patient and carer participants to ensure the representation of participants’ perspectives. However, settings were geographically limited, it was not possible to involve all key stakeholder groups, and the full extent of experience of using digital advance care planning systems may not be reflected in the data. The use of the NASSS framework helped identify and understand uncertainties and interdependencies but orientated the reporting towards a problem rather than a solution-focussed narrative.

## Conclusion

The implementation of digital advance care planning systems can be conceptualised in five components: sociocultural, technical and structural prerequisites; recognition of clinical need for conversation and digital advance care planning; having conversations and documenting decisions; accessing, actioning and amending; and using data to support evaluation and implementation. Patients, carers, professionals and commissioners hold varying and sometimes uncertain views on what digital advance care planning system are, who they are for, their purpose and how they should be evaluated. Breakdowns in the technological, infrastructure and human factor influences related to digital advance care planning systems risk undermining the safety and quality of patient care and increasing the risk of unintended consequences. To optimise digital advance care planning system implementation, amendments to technical features of systems must consider wider technological, infrastructure and human factor influences.

## Supplemental Material

sj-docx-1-pmj-10.1177_02692163241280134 – Supplemental material for Exploring the contextual assumptions, interventions and outcomes of digital advance care planning systems: A theory of change approach to understand implementation and evaluation Supplemental material, sj-docx-1-pmj-10.1177_02692163241280134 for Exploring the contextual assumptions, interventions and outcomes of digital advance care planning systems: A theory of change approach to understand implementation and evaluation by Andy Bradshaw, Matthew J Allsop, Jacqueline Birtwistle, Catherine J Evans, Samuel D Relton, Suzanne H Richards, Maureen Twiddy, Robbie Foy, Pablo Millares Martin, Sarah Yardley and Katherine E Sleeman in Palliative Medicine

sj-docx-2-pmj-10.1177_02692163241280134 – Supplemental material for Exploring the contextual assumptions, interventions and outcomes of digital advance care planning systems: A theory of change approach to understand implementation and evaluation Supplemental material, sj-docx-2-pmj-10.1177_02692163241280134 for Exploring the contextual assumptions, interventions and outcomes of digital advance care planning systems: A theory of change approach to understand implementation and evaluation by Andy Bradshaw, Matthew J Allsop, Jacqueline Birtwistle, Catherine J Evans, Samuel D Relton, Suzanne H Richards, Maureen Twiddy, Robbie Foy, Pablo Millares Martin, Sarah Yardley and Katherine E Sleeman in Palliative Medicine
