# Supplementary material for: Exploring the contextual assumptions, interventions and outcomes of digital advance care planning systems: A theory of change approach to understand implementation and evaluation
Source: Palliat Med. 2024 Sep 21;38(10):1144–55. doi: 10.1177/02692163241280134 (PMC11613644; doi:10.1177/02692163241280134)
Supplement: sj-docx-1-pmj-10.1177_02692163241280134 – Supplemental material for Exploring the contextual assumptions, interventions and outcomes of digital advance care planning systems: A theory of change approach to understand implementation and evaluation [file sj-docx-1-pmj-10.1177_02692163241280134.docx]

**Appendix A:** Examples of event artefacts generated during workshops 1 and 2

**A
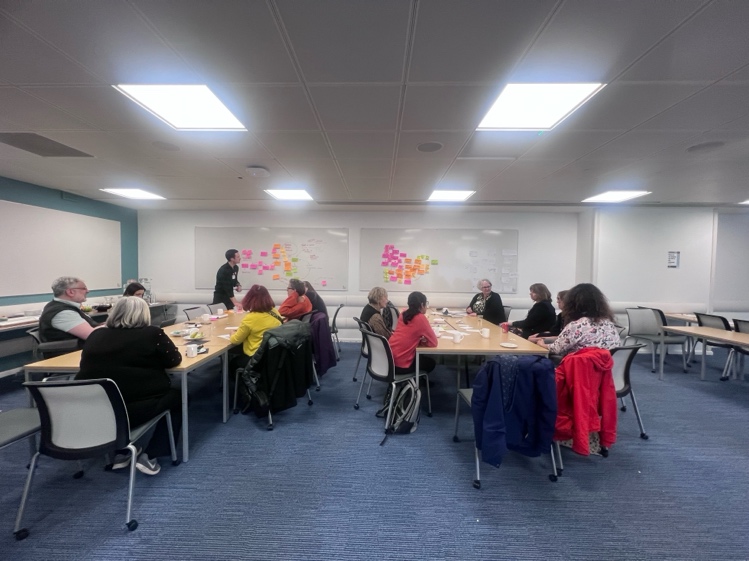
 B
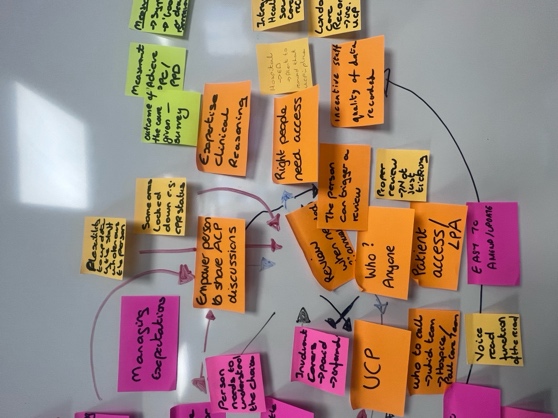
**

**C
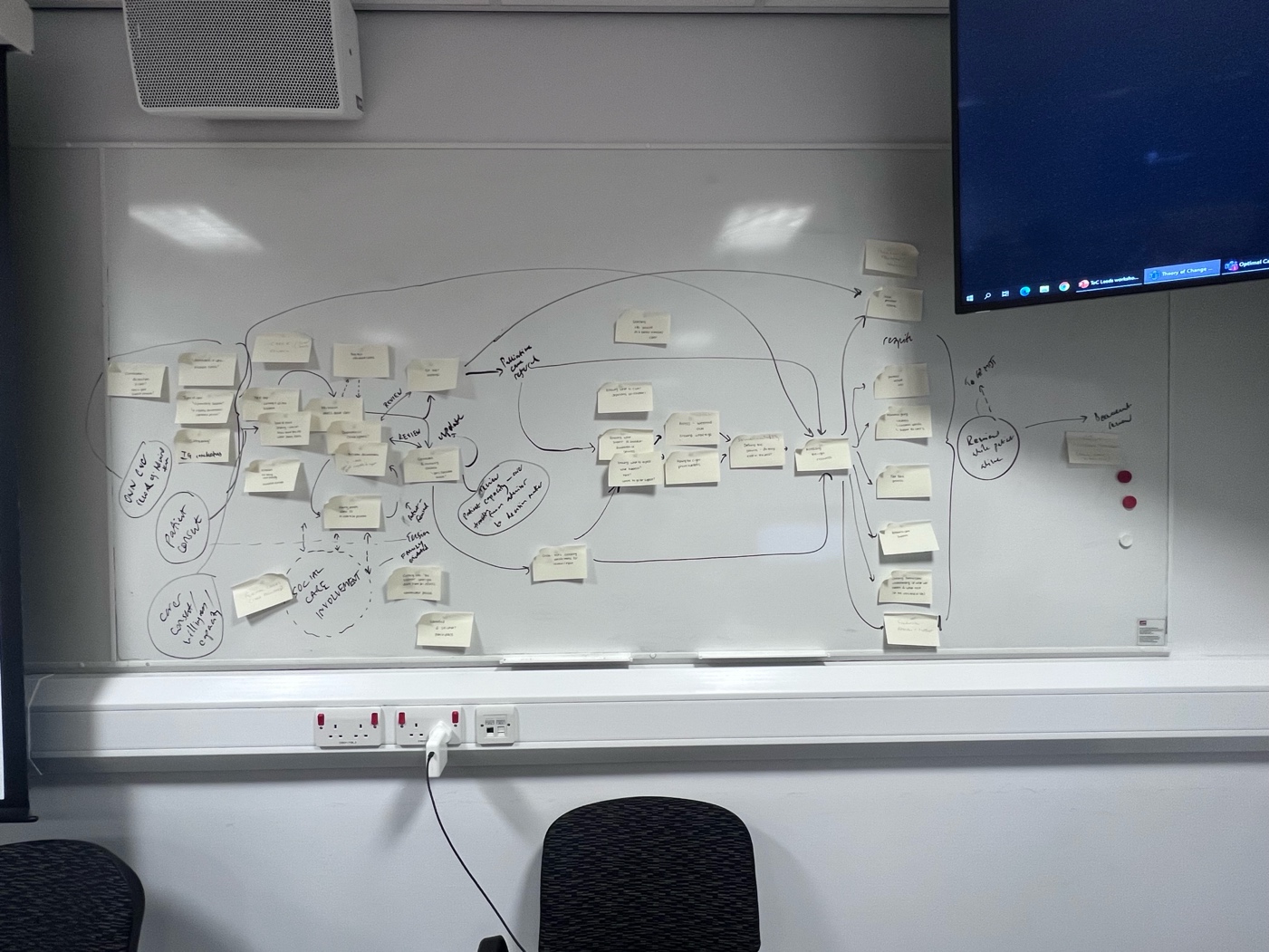
**

**D
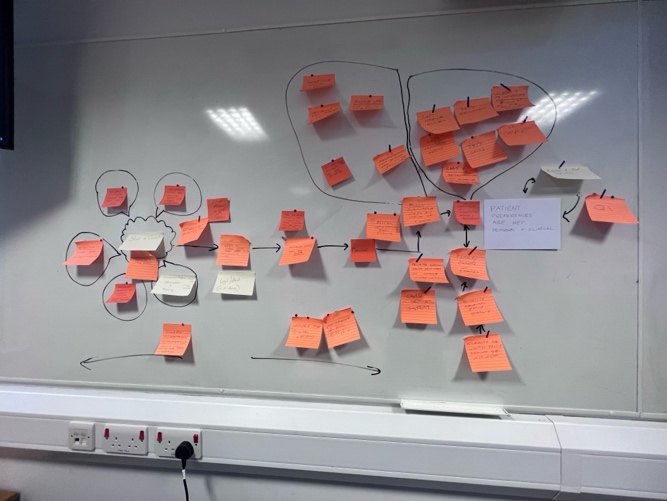
 E
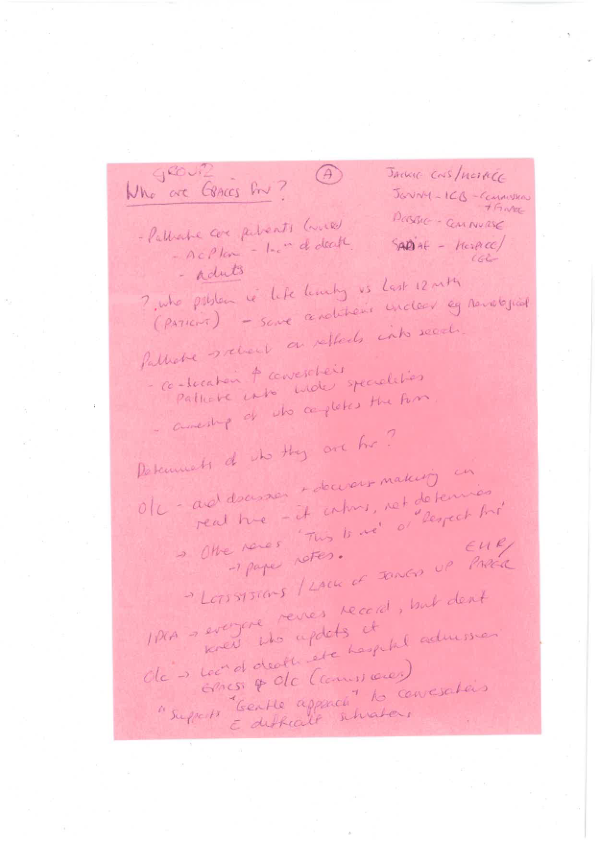
**

*Key: Examples of event artefacts include photographs of workshop activities (A), photographs and sticky notes of Theory of Change maps generated during the workshops (B, C, D) and researcher field notes scribed during the workshops (E).*
