# Supplementary material for: Exploring the contextual assumptions, interventions and outcomes of digital advance care planning systems: A theory of change approach to understand implementation and evaluation
Source: Palliat Med. 2024 Sep 21;38(10):1144–55. doi: 10.1177/02692163241280134 (PMC11613644; doi:10.1177/02692163241280134)
Supplement: sj-docx-2-pmj-10.1177_02692163241280134 – Supplemental material for Exploring the contextual assumptions, interventions and outcomes of digital advance care planning systems: A theory of change approach to understand implementation and evaluation [file sj-docx-2-pmj-10.1177_02692163241280134.docx]

**Appendix B:** Quality criteria selected for the study

The table below outlines the quality criteria selected for this study, drawing on contemporary work and thinking on rigour in qualitative research.^32, 36-40^ The table also details how criteria were fulfilled throughout the research process.

| **Quality criteria** | **How it was fulfilled in this study** |
| --- | --- |
| **Sincerity** (did the research team engage in reflexivity and were they transparent about the research process?) | We have been transparent in outlining each step of data collection and analysis. The research team engaged in different forms of reflexivity, including reflecting introspectively (inward reflections on how their own biases, experiences, and histories impacted the research process and vice versa) and intersubjectively (reflections on relationships between the them and participants). ^41^  Analysis and write-up was a collaborative process. PPI members and members of the wider interdisciplinary research team acted as ‘critical friends’. ^40^ This entailed working collaboratively through regular meetings and written feedback in which findings were constructively challenged, reflexivity encouraged, and alternative interpretations of the data proposed. |
| **Width** (how comprehensive is the evidence provided?) | Data that was collected over two UK regions (West Yorkshire and London), from 38 participants. Participants included patients with different types of life-limiting illnesses, carers with different caring experiences, end-of-life commissioners, and health and care professionals of different roles who worked across different settings of care. |
| **Exploiting exceptional data** (were contradictory data attended to during analysis?) | Our data was complex and consisted of a mixture of complementary, unique, and sometimes contradictory viewpoints. The NASSS framework to attend to and incorporate contradictory, divergent, and non-dominant (or ‘deviant’/’exceptional’) data into our findings. |
| **Credibility** (have thick descriptions and detailed findings been provided?) | Data has been integrated from across three different workshops. Through adopting a pluralistic approach to data analysis and attending to outliers we have provided a well-rounded and comprehensive (i.e., ‘thick’) description of findings. |
| **Resonance** (are the findings of the research generalisable or transferable?) | Through providing thick description of study findings, drawing on theory, and including a wide sample, readers are likely able to make making generalisations based on the transferability of findings to similar contexts and their resonance with personal experiences. |
| **Meaningful coherence** (does the study and analysis achieve its intended goals through using appropriate methods?) | This study aimed to understand participants’ views on EPaCCS, how they were being used in everyday practice, alongside how their uptake and implementation may be optimised. The methods used for data collection, analysis, write-up, and ensuring rigour were appropriate in achieving these. |
| **Substantive contribution** (Does this piece contribute to understandings on the topic of investigation) | This is the first study of its kind to combine a Theory of Change approach with the NASSS framework to provide a rich and detailed account of how EPaCCS is viewed, engaged with, and implemented in the practice by patients, carers, and professionals. |
